# Bevacizumab plus capecitabine and cisplatin in Chinese patients with inoperable locally advanced or metastatic gastric or gastroesophageal junction cancer: randomized, double-blind, phase III study (AVATAR study)

**DOI:** 10.1007/s10120-014-0351-5

**Published:** 2014-02-21

**Authors:** Lin Shen, Jin Li, Jianming Xu, Hongming Pan, Guanghai Dai, Shukui Qin, Liwei Wang, Jinwan Wang, Zhenzhou Yang, Yongqian Shu, Ruihua Xu, Lei Chen, Yunpeng Liu, Shiying Yu, Lilian Bu, Yongzhe Piao

**Affiliations:** 1 Peking University Cancer Hospital and Institute, No 52 Fucheng Road, Haidian District, Beijing, China; 2 Fudan University Cancer Hospital, Shanghai, China; 3 Beijing 307 Hospital, Beijing, China; 4 Shao Yifu Hospital, Hangzhou, China; 5 Beijing 301 Hospital, Beijing, China; 6 Nanjing Bayi Hospital, Nanjing, China; 7 Shanghai First People’s Hospital, Shanghai, China; 8 CAMS Cancer Hospital, Beijing, China; 9 Cancer Center, Research Institute of Surgery, Daping Hospital, Third Military Medical University, Chongqing, China; 10 Jiangsu Renmin Hospital, Nanjing, China; 11 Zhongshan University Cancer Hospital, Guangzhou, China; 12 Shantou Medical University Cancer Hospital, Shantou, China; 13 1st Affiliated Hospital of China Medical University, Shenyang, China; 14 Tongji Hospital, Tongji Medical College of Huazhong University of Science and Technology, Wuhan, China; 15 Roche Product Development in Asia Pacific, Shanghai, China

**Keywords:** Bevacizumab, Gastric adenocarcinoma

## Abstract

**Background:**

In the AVAGAST study, fluoropyrimidine and cisplatin plus bevacizumab did not significantly improve overall survival (OS) versus fluoropyrimidine and cisplatin plus placebo in patients with advanced gastric cancer. Geographic differences in efficacy were observed in AVAGAST, but the study only included 12 Chinese patients. AVATAR, a study similar in design to AVAGAST, was a randomized, double-blind, phase III study conducted in Chinese patients with advanced gastric cancer.

**Methods:**

Patients more than 18 years of age with gastric adenocarcinoma were randomized 1:1 to capecitabine–cisplatin plus either bevacizumab or placebo. The primary endpoint was OS; secondary endpoints included progression-free survival (PFS) and safety.

**Results:**

In total, 202 patients were included (placebo *n* = 102; bevacizumab *n* = 100). Baseline characteristics were well balanced. The primary analysis result did not show a difference in OS for the bevacizumab arm compared to the placebo arm [hazard ratio, 1.11 (95 % CI, 0.79–1.56); *P* = 0.5567]. Median PFS was also similar in both arms. Bevacizumab plus capecitabine–cisplatin was well tolerated. Grade 3–5 adverse events (AEs) occurred in 60 % of bevacizumab-treated and 68 % of placebo-treated patients, respectively. Grade 3–5 AEs of special interest with bevacizumab occurred in 8 % of bevacizumab-treated patients and 15 % of placebo-treated patients, mainly grade 3–5 hemorrhage (bevacizumab 4 %, placebo 12 %).

**Conclusions:**

Addition of bevacizumab to capecitabine–cisplatin in Chinese patients with advanced gastric cancer did not improve outcomes in AVATAR. There was no difference in OS between the two arms and PFS was similar in both arms. Safety findings were as previously experienced with bevacizumab, including AVAGAST; no new safety signals were reported.

## Introduction

Gastric cancer is the third most commonly diagnosed cancer and the second most common cause of cancer-related deaths worldwide [1]. In China, gastric cancer is one of the most common malignancies, ranking second in incidence and third in mortality [2]. In 2008, there were 989,000 new cases of gastric cancer and 737,000 deaths worldwide; of these, 464,000 new cases (47 %) and 352,000 deaths (48 %) occurred in China [2]. To date, surgery is still the only curative treatment for patients with gastric cancer, but this is only an option for patients with early or locally advanced gastric cancer. At present, the early-stage diagnosis rate is low in China, and as a result, most patients have advanced or metastatic gastric cancer at diagnosis.

The only treatment option for patients with advanced gastric cancer is chemotherapy, although the efficacy of such treatment is limited [3–5]. At present, the most widely used treatment consists of a fluoropyrimidine and a platinum compound. The combination of capecitabine with cisplatin has demonstrated non-inferiority in terms of efficacy and a similar safety profile compared with 5-fluorouracil (5-FU) plus cisplatin [6]. As a result, capecitabine is considered an attractive alternative to intravenous 5-FU.

Angiogenesis is regulated by a balance between local pro-angiogenic and anti-angiogenic factors. Vascular endothelial growth factor (VEGF) is the most potent and specific promoter of angiogenesis and is a key physiologic regulator of new vessel formation during embryogenesis, skeletal growth, and reproductive functions. It is also implicated in pathologic angiogenesis, such as that associated with tumor growth [7]. VEGF expression is strongly correlated with tumor progression and poor prognosis in many tumors, including gastric cancer [8–12].

Bevacizumab is a humanized monoclonal antibody that blocks the binding of human VEGF to its receptors. Clinical data have shown that bevacizumab can be combined with a range of cytotoxic and other anticancer agents for the treatment of a variety of solid tumors [13–16]. A nonrandomized phase II study of bevacizumab in combination with irinotecan and cisplatin in 47 patients with advanced gastric cancer showed promising efficacy compared with historical controls, without an unacceptable increase in thromboembolic events, gastrointestinal perforation, or bleeding [17]. In addition, phase III trials have shown superior efficacy with manageable toxicity when bevacizumab was given in combination with chemotherapy to patients with advanced colorectal or lung cancers [13, 14].

These data strongly supported further exploration of this approach in patients with gastric cancer. Therefore, the randomized phase III AVATAR study was undertaken to investigate the possible benefit of adding bevacizumab to first-line chemotherapy in Chinese patients with advanced gastric cancer. At the time of initiating the AVATAR study, the global AVAGAST study was ongoing [18].

## Methods

### Study design and patient population

AVATAR was a randomized, double-blind, multicenter, phase III trial conducted in 14 hospitals in China (www.clinicaltrials.gov identifier NCT00887822). The study was conducted in accordance with Good Clinical Practice guidelines and the Declaration of Helsinki. All patients provided written informed consent. Approvals for the study protocol (and any modifications thereafter) were obtained from independent ethics committees at study centers.

Patients were more than 18 years of age, with histologically confirmed, inoperable, locally advanced or recurrent, and/or metastatic adenocarcinoma of the stomach or gastroesophageal junction. Patients with no prior treatment for advanced/metastatic disease, Eastern Cooperative Oncology Group (ECOG) performance status 0–2, adequate organ function, and measurable or nonmeasurable but evaluable disease were included in the study. Pregnant or lactating women were excluded from the study. Other major exclusion criteria included severe cardiovascular disease, lack of physical integrity of the upper gastrointestinal tract or malabsorption syndrome, active gastrointestinal bleeding, and evidence of brain metastases.

### Procedures

This was a double-blind study in which neither patients nor investigators knew which treatment patients were receiving. Patients who were eligible for study entry were randomly assigned (1:1) to one of the two treatment groups via an interactive voice response system using the dynamic least-squares minimization randomization method. Randomization was stratified according to ECOG performance status (0/1 or 2) and disease status (locally advanced or metastatic).

Bevacizumab 7.5 mg/kg or placebo (bevacizumab vehicle) was given by intravenous infusion on day 1 every 3 weeks until disease progression, unacceptable toxicity, or withdrawal of consent. Capecitabine 1,000 mg/m^2^ was given orally twice daily for 14 days, followed by a 1-week rest, until disease progression, unacceptable toxicity, or withdrawal of consent. Cisplatin 80 mg/m^2^ was given by intravenous infusion on day 1 every 3 weeks for six cycles. Chemotherapy dose adjustments were allowed. Bevacizumab toxicity was managed by treatment interruptions. Crossover to bevacizumab at the time of disease progression was not allowed.

### Assessments

Medical history, chest X-ray, and electrocardiogram (ECG) were performed within 21 days before randomization. Assessments of vital signs, ECOG performance status, creatinine clearance, and a routine blood analysis (hematology and chemistry) were performed within 7 days of randomization. During the treatment period, physical examination, hematology, biochemistry, and urinalysis were repeated at the beginning of each cycle.

Tumor assessments (computed tomography/magnetic resonance imaging) were performed within 21 days before randomization, and were repeated every 6 weeks for the first year after randomization and every 12 weeks thereafter until disease progression. Tumor response was evaluated by investigators using Response Evaluation Criteria in Solid Tumors (version 1.0). Survival status was monitored during the treatment period and every 3 months after treatment completion until death.

Adverse events and serious adverse events were assessed according to the National Cancer Institute Common Terminology Criteria for Adverse Events (version 3.0) according to International Conference on Harmonisation guidelines.

### Statistical analysis

Efficacy analysis was primarily based on the intent to treat (ITT) population, which population included all patients randomized during the study. All patients who were randomized and received at least one dose/infusion of any component of study medication were included in the safety population.

The planned sample size was 200 patients, which took into account two points. First, during the time of planning the AVATAR study, the multiregional phase III AVAGAST trial was ongoing. Using the AVAGAST target hazard ratio (HR) of 0.78 and the bridging study concept, simulated results were obtained based on the following assumptions: recruitment period of 17 months; study continued up to a maximum of 18 months after last patients was randomized or 60 % of patients reached the study primary endpoint (death from any cause), whichever occurred first; median OS of 10 months in the control arm and 12.8 months in the experimental arm; 5 % dropout rate; and would show that the 200 patients had ≥80 % probability of demonstrating a positive treatment effect (i.e., HR < 1, showing a trend toward efficacy) for the study. Second, 100 were required in each treatment arm to satisfy Chinese Regulatory Agency requirements for a safety population.

The primary endpoint was overall survival, defined as the time between date of randomization and date of death from any cause. Secondary endpoints were progression-free survival, defined as the time from the date of randomization until the day of documented disease progression or death from any cause, whichever occurred earlier, and response rate. Tumor assessment was performed using Response Evaluation Criteria in Solid Tumors (version 1.0).

An unstratified log-rank test was used to compare survival functions between the two treatment groups. Kaplan–Meier methodology was used to estimate the median overall survival for each treatment group. Estimates of the treatment effect were expressed as HRs through use of Cox regression analyses.

The final analysis was planned when 120 deaths had occurred. Pre-planned analyses of overall survival using Cox’s proportional hazards models were conducted with the stratification variables and other relevant covariates (ECOG performance status, prior (neo)adjuvant chemotherapy, sex, age, disease status, number of baseline metastatic sites, prior gastrectomy, liver metastases, and bone metastasis at baseline).

## Results

### Patients

**Fig. 1:**
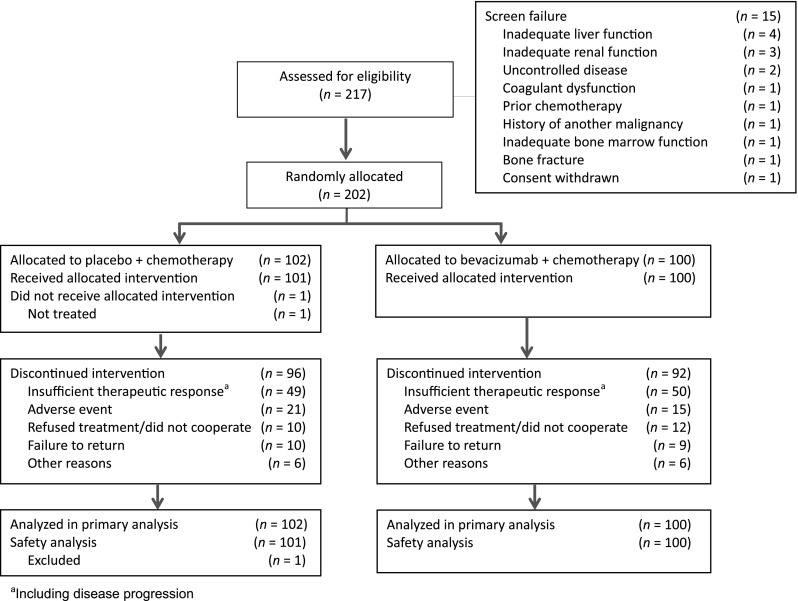
CONSORT diagram

Between March 25, 2009, and July 12, 2010, 202 patients recruited from 14 sites were randomized to capecitabine–cisplatin plus either placebo (*n* = 102) or bevacizumab (*n* = 100; Fig. 1). One patient did not receive any study drug and was excluded from the safety analysis. Patient demographics and baseline characteristics of the two treatment arms were generally well balanced for the ITT population (Table 1).

**Table 1:** Patient demographics and baseline characteristics (intent to treat population)

Characteristic	Placebo + capecitabine–cisplatin (*n* = 102)	Bevacizumab + capecitabine–cisplatin (*n* = 100)
Male, *n* (%)	74 (72.5)	68 (68.0)
Mean age, years	55.5	54.2
Age category, *n* (%)		
<40 years	11 (10.8)	14 (14.0)
40–65 years	66 (64.7)	67 (67.0)
≥65 years	25 (24.5)	19 (19.0)
Eastern Cooperative Oncology Group performance status, *n* (%)		
0/1	97 (95.1)	95 (95.0)
≥2	5 (4.9)	5 (5.0)
Disease status, *n* (%)		
Locally advanced	8 (7.8)	5 (5.0)
Metastatic	94 (92.2)	95 (95.0)
Primary site, *n* (%)		
Stomach	82 (80.4)	85 (85.0)
Gastroesophageal junction	20 (19.6)	15 (15.0)
Measurable disease, *n* (%)	86 (84.3)	81 (81.0)
Staging, *n* (%)		
III	4 (3.9)	4 (4.0)
IV	98 (96.1)	96 (96.0)
Type of gastric cancer, *n* (%)		
Adenocarcinoma	94 (92.1)	95 (95.0)
Signet-ring cell carcinoma	8 (7.8)	4 (4.0)
Squamous cell carcinoma	0 (0.0)	1 (1.0)
Adenocarcinoma differentiation status, *n* (%)		
Well differentiated	2 (2.0)	2 (2.0)
Moderately differentiated	17 (16.7)	15 (15.0)
Poorly differentiated	48 (47.1)	51 (51.0)
Unknown differentiated	35 (34.3)	31 (31.0)
Prior adjuvant/neoadjuvant chemotherapy, *n* (%)	7 (6.9)	10 (10)
Prior gastrectomy, *n* (%)	20 (19.6)	24 (24)
Number of metastatic sites at baseline, *n* (%)		
≤1	59 (57.8)	60 (60.0)
≥2	43 (42.2)	40 (40.0)
Liver metastasis, *n* (%)	40 (39.2)	39 (39.0)
Bone metastases, *n* (%)	3 (2.9)	4 (4.0)

### Efficacy

At data cutoff (May 13, 2011), 131 deaths had occurred (63 in the placebo arm and 68 in the bevacizumab arm). The median duration of treatment was 4.8 months in the placebo arm and 4.4 months in the bevacizumab arm. The median duration of follow-up was 10.5 months in the placebo arm and 10.0 months in the bevacizumab arm. Posttreatment nonstudy therapies for gastric cancer after disease progression were reported for 15 of 102 patients (15 %) in the placebo arm and 11 of 100 patients (11 %) of patients in the bevacizumab arm, with the majority receiving chemotherapy [placebo: 13 % (13/102 patients); bevacizumab: 9 % (9/100 patients)]. The most commonly used chemotherapy agents (≥5 % in either treatment arm) were antineoplastic agents (placebo: 9 % vs. bevacizumab: 6 %) and taxanes (6 % vs. 2 %, respectively).

**Fig. 2:**
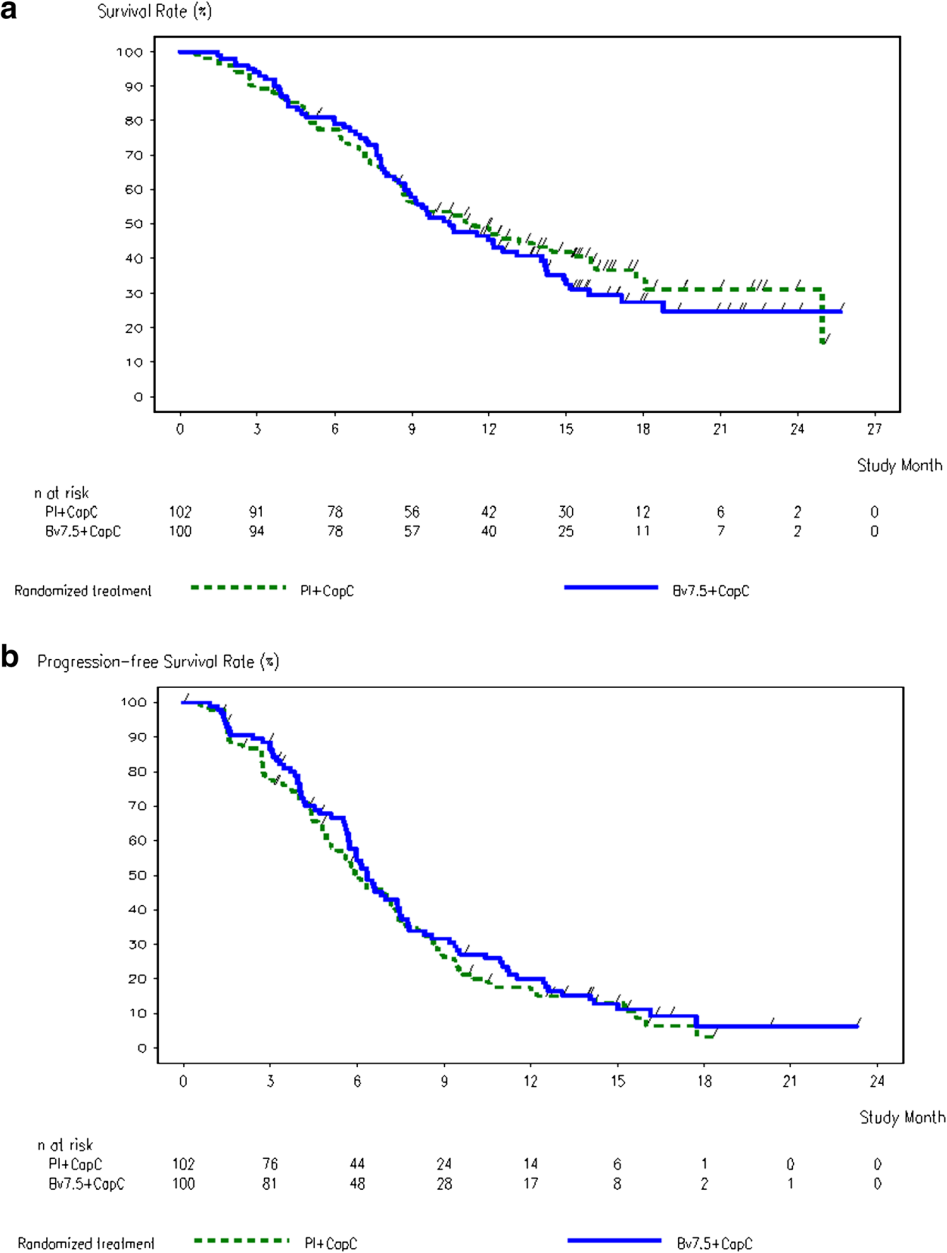
Kaplan–Meier curves for overall (**a**) and progression-free (**b**) survival in patients treated with placebo plus chemotherapy or bevacizumab plus chemotherapy (intent to treat population)

Median overall survival was 11.4 months [95 % confidence interval (CI), 8.6–16.0 months] in the placebo arm versus 10.5 months (8.9–14.1 months) in the bevacizumab arm. There was no statistically significant difference in overall survival between treatment arms [HR, 1.11 (95 % CI, 0.79–1.56); *P* = 0.56] (Table 2; Fig. 2a). The 1-year survival rate was 48 % in the placebo arm and 45 % in the bevacizumab arm. Median progression-free survival was 6.0 months (95 % CI, 4.9–7.4 months) in the placebo arm versus 6.3 months (95 % CI, 5.7–7.4 months) in the bevacizumab arm [HR 0.89 (95 % CI, 0.66–1.21), *P* = 0.47] (Table 2; Fig. 2b).

**Table 2:** Analysis of efficacy (intent to treat population)

Outcome	Placebo + capecitabine–cisplatin (*n* = 102)	Bevacizumab + capecitabine–cisplatin (*n* = 100)
Overall survival		
Patients with event, *n* (%)	63 (61.8)	68 (68.0)
Median overall survival (95 % CI), months	11.4 (8.6–16.0)	10.5 (8.9–14.1)
Unadjusted hazard ratio (95 % CI)	1.11 (0.79–1.56)	
*P* value*	0.5567	
Progression-free survival		
Patients with event, *n* (%)	83 (81.4)	81 (81.0)
Median progression-free survival (95 % CI), months	6.0 (4.9–7.4)	6.3 (5.7–7.4)
Unadjusted hazard ratio (95 % CI)	0.89 (0.66–1.21)	
*P* value^a^	0.4709	
Overall response during first-line therapy (investigator evaluation)	(*n* = 86)	(*n* = 81)
Responders, *n* (%)	29 (33.7)	33 (40.7)
Complete response	1 (1.2)	0 (0.0)
Partial response	28 (32.6)	33 (40.7)
Stable disease	33 (38.4)	28 (34.6)
Progressive disease	11 (12.8)	7 (8.6)
Missing (no response assessment)	13 (15.1)	13 (16.0)
Difference in response rates, % (95 % CI)	7.02 (−8.3 to 22.4)	
*P* value^b^	0.3480	

*CI* confidence interval

^a^Log-rank test

^b^χ^2^ test

The proportion of patients with a response to treatment (confirmed complete or partial response) was numerically higher in the bevacizumab arm compared with the placebo arm, but this difference did not reach statistical significance [bevacizumab, 33 of 81 patients (41 %) vs. placebo, 29 of 86 patients (34 %), *P* = 0.35] (Table 2).

Subgroup analyses were performed for overall survival. The estimated HRs in most subgroups were about 1.00, and all CIs included 1.00.

### Safety

The majority of patients in each treatment arm experienced at least one adverse event. Vomiting, nausea, neutropenia, and anorexia were the most common adverse events in both arms. The incidence of grade 3–5 adverse events was similar in the two arms [*n* = 69 (68 %) in the placebo arm vs. *n* = 60 (60 %) in the bevacizumab arm]. The incidence of grade 3–5 adverse events of special interest with bevacizumab was higher in the placebo arm (*n* = 15, 15 %) than in the bevacizumab arm (*n* = 8, 8 %) (Table 3). Hemorrhage was the most common grade 3–5 adverse event, with a higher incidence in the placebo arm (*n* = 12, 12 %) than in the bevacizumab arm (*n* = 4, 4 %). Adverse events leading to death occurred in eight patients (8 %) in the placebo arm and four patients (4 %) in the bevacizumab arm.

**Table 3:** Most common grade 3–5 adverse events and adverse events of special interest with bevacizumab (related and unrelated events; safety population)

Event, *n* (%)	Placebo + capecitabine–cisplatin (*n* = 101)	Bevacizumab + capecitabine–cisplatin (*n* = 100)
Any grade 3–5 adverse events	69 (68.3)	60 (60.0)
Vomiting	10 (9.9)	22 (22.0)
Neutropenia	18 (17.8)	14 (14.0)
Nausea	6 (5.9)	9 (9.0)
Anemia	5 (5.0)	5 (5.0)
Intestinal obstruction	3 (3.0)	5 (5.0)
Decreased appetite	1 (1.0)	5 (5.0)
Leukopenia	9 (8.9)	4 (4.0)
Palmar–plantar erythrodysesthesia syndrome	6 (5.9)	4 (4.0)
Thrombocytopenia	2 (2.0)	4 (4.0)
Hypokalemia	3 (3.0)	3 (3.0)
Lung infection	0	3 (3.0)
Abdominal pain	1 (1.0)	3 (3.0)
Diarrhea	3 (3)	2 (2.0)
Gastrointestinal hemorrhage	1 (1.0)	2 (2.0)
Hyperbilirubinemia	0	2 (2.0)
Mouth ulceration	0	2 (2.0)
Cerebral infarction	0	2 (2.0)
Upper gastrointestinal hemorrhage	8 (7.9)	1 (1.0)
Hyponatremia	3 (3.0)	1 (1.0)
Any grade 3–5 events of special interest with bevacizumab	15 (14.9)	8 (8.0)
Hemorrhage	12 (11.9)	4 (4.0)
Arterial thromboembolic events	4 (4.0)	3 (3.0)
Venous thromboembolic events	1 (1.0)	1 (1.0)
Hypertension	1 (1.0)	0 (0.0)
Gastrointestinal perforation	0 (0.0)	1 (1.0)

## Discussion

Despite extensive evaluation of multiple chemotherapy regimens, no international consensus exists regarding the optimal first-line treatment regimen for patients with advanced gastric cancer. In Western countries and in Asia, the standard chemotherapy regimen for first-line treatment of metastatic gastric cancer consists of a fluoropyrimidine (5-FU or capecitabine) in combination with a platinum agent (cisplatin or oxaliplatin) with or without a third cytotoxic drug (usually epirubicin or docetaxel) [5]. AVAGAST, which was the first phase III study to evaluate bevacizumab in combination with chemotherapy for the first-line treatment of patients with advanced gastric cancer, did not reach its primary endpoint of an HR of 0.87 for overall survival in the overall study population. Subgroup analyses, however, suggested regional differences in efficacy, with a greater benefit being seen in the European and Pan-American regions. In contrast, there was no benefit for bevacizumab in Asian patients, 90 % of whom were recruited from Japan and Korea (Table 4) [18].

**Table 4:** Baseline characteristics and efficacy of AVAGAST and AVATAR by region (intent to treat populations)

	AVAGAST study						AVATAR study		ToGA study	
	Asia		Europe		Pan-America		China		China subgroup	
	BEV (*n* = 188)	Placebo (*n* = 188)	BEV (*n* = 125)	Placebo (*n* = 124)	BEV (*n* = 74)	Placebo (*n* = 75)	BEV (*n* = 100)	Placebo (*n* = 102)	Trastuzumab (*n* = 36)	Placebo (*n* = 48)
Male (%)	68	67	66	67	64	65	68	73	78	81
Median age (years)	58.5	59.0	59.0	59.0	53.5	56.0	56	59	58.7	58.2
ECOG PS (%)										
0/1	98	95	89	93	95	97	95	95	81	81
2	2	5	11	7	5	3	5	5	19	19
Primary tumor site GEJ (%)	7	5	23	22	15	17	15	20	19	21
Measurable disease (%)	76	70	87	89	81	73	81	84	89	90
Liver metastases (%)	29	26	35	38	42	41	39	39	53*	44^a^
Prior gastrectomy (%)	32	31	22	25	31	23	24	20	14	6
Further treatment (%)	59	67	24	29	24	15	11	15	11	10
Median overall survival (months)	13.9	12.1	11.1	8.6	11.5	6.8	10.5	11.4	12.6	9.7
HR (95 % CI)	0.97 (0.75–1.25)		0.85 (0.63–1.14)		0.63 (0.43–0.94)		1.11 (0.79–1.56)		0.72 (0.40–1.29)	
Median progression-free survival (months)	6.7	5.6	6.9	4.4	5.9	4.4	6.3	6.0	6.8	5.5
HR (95 % CI)	0.92 (0.74–1.14)		0.71 (0.54–0.93)		0.65 (0.46–0.93)		0.89 (0.66–1.21)		0.69 (0.41–1.15)	
ORR (%)	47.9	45.5	41.3	28.2	50.0	36.4	40.7	33.7	36.1	33.3
OR (95 % CI)	1.10 (0.69–1.77)		1.79 (1.02–3.15)		1.75 (0.83–3.69)		1.19 (0.65–2.20)		1.13 (0.46–2.80)	

*BEV* bevacizumab, *CI* confidence interval, *ECOG* Eastern Cooperative Oncology Group, *GEJ* gastroesophageal junction, *HR* hazard ratio, *ORR* overall response rate, *OR* odds ratio, *PS* performance status

^a^Organ (lung or liver) with metastases

Similar to the Asian subgroup data reported for AVAGAST, the AVATAR study did not show an improvement in overall survival for patients treated with bevacizumab plus capecitabine–cisplatin compared with placebo plus capecitabine–cisplatin (HR, 1.11). Progression-free survival was also similar in both treatment arms, and although a numerically higher response rate was observed in bevacizumab-treated patients, this difference did not reach statistical significance.

The design of the AVATAR study was similar to that of AVAGAST, although there were different prognosis patterns at baseline in both studies (Table 4). Specifically, patients in AVATAR differed from Asian patients in AVAGAST, the latter being mainly from Japan and Korea, in that they had a greater incidence of having liver metastases and gastroesophageal junction tumors and less frequently had a prior gastrectomy. Another remarkable finding is our patients were less likely to receive a second and further line of therapy after disease progression because medical insurance in China does not cover second-line drugs. Overall, our patients were more comparable to the European and Pan-American patients in AVAGAST than the Asian subgroup. Accordingly, the better outcome of European and Pan-American subgroup in AVAGAST study may difficult to explain by the different second- and further line treatment rate across geographic regions. Notably, the subgroup of Chinese patients in the ToGA study also had comparable demographic and disease characteristics with those of the AVATAR study population (Table 4) [19]. The heterogeneity between Western and Asian populations, as well as between countries, needs further investigation; at present, no clear conclusions can be drawn from these data.

Bevacizumab in combination with capecitabine and cisplatin was well tolerated by the Chinese patients in the present study. The safety profile was generally consistent with previous experience with bevacizumab in other indications and no new safety signals were observed. Of note, AEs of special interest to bevacizumab were more common in the placebo arm; this was mainly the result of a higher incidence of hemorrhage, but additional medical review of the data did not clearly identify any reasons for this finding.

Overall, the AVATAR study failed to show any efficacy advantage for the addition of bevacizumab to chemotherapy in Chinese patients with advanced/metastatic gastric cancer: there was no difference in overall survival or progression-free survival between the bevacizumab and placebo arms in this population. The safety findings in AVATAR were similar to previous reports, and no new safety signals were reported. The limitation of the present study is the lack of combined pharmacokinetic data, as well as the lack of biomarker research.

In conclusion, addition of bevacizumab to capecitabine–cisplatin in Chinese patients with advanced gastric cancer did not improve outcomes in AVATAR. There was no difference in OS between patients treated with capecitabine–cisplatin plus either bevacizumab or placebo, and PFS was similar in both arms. Safety findings were as previously experienced with bevacizumab, including AVAGAST; no new safety signals were reported.
